# Preparation supported heteropoly (acid)/polyaniline catalysts and catalytic synthesis of tributyl citrate

**DOI:** 10.1039/c9ra06071e

**Published:** 2019-10-16

**Authors:** Limin Wang, Bin Ding, Miao Zhang

**Affiliations:** Shandong Applied Research Center of Nanogold Technology (Au-SDARC), School of Chemistry & Chemical Engineering, Yantai University Yantai 264005 China zhangmiao@ytu.edu.cn +86 535 6911732 +86 535 6911732; Institute of Petrochemical Technology, Jilin Institute of Chemical Technology Jilin 132022 China

## Abstract

A series of polyaniline supported heteropoly acids were prepared through a simple method at room temperature. The obtained heterogeneous catalysts were comprehensively characterized by powder FTIR spectroscopy, UV-vis spectra, NH_3_ temperature programmed desorption (TPD) and scanning electron microscopy (SEM). The influence of various process parameters such as heteropoly loading (10 to 25 wt%), catalyst amount (3–5%), molar ratio of *n*-butanol to citric acid (3 to 5), and reaction time (3.5–12 h) have been investigated over heteropoly/polyaniline catalysts with the aim to maximize citric acid conversion and tributyl citrate selectivity. The different catalytic tests has shown that the catalyst exhibits high conversion and selectivity by using the as-prepared heteropoly/polyaniline catalysts for esterification under appropriate conditions. The present method of using 20% heteropoly/polyaniline catalyst for the synthesis of tributyl citrate would be environmentally benign in the reusability of catalyst.

## Abstract 2

A series of polyaniline supported heteropoly acids were prepared through a simple method at room temperature.

## Graphical abstract

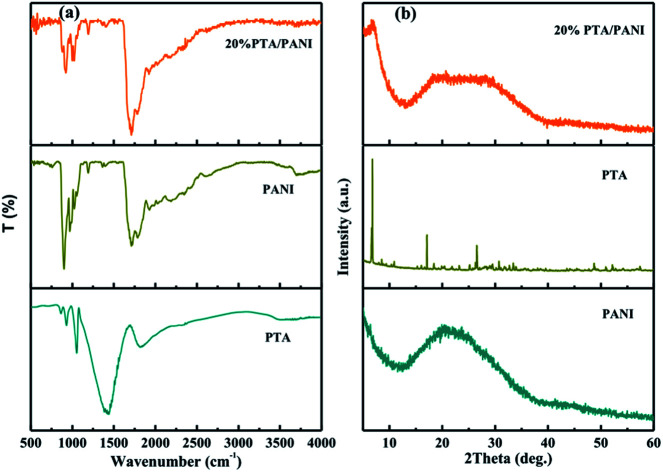

## Introduction

Recently, the plasticizers market is growing worldwide, as projected by latest statistical data at about 11 billion pounds per year of which, 2.4 billion pounds is shared by the United States.^1^ Tributyl citrate (TBC) has been applied in polymer industries such as solvents for synthesis of polyvinyl chloride (PVC) and its copolymers.^2^ The copolymers of TBC are widely applied in food wrapping films because TBC is a vital thermally stable and non-toxic plasticizer.^3^ TBC does not discolour when processed in compounded resins because of the excellent thermal stability of TBC.^4^ TBC as a non-toxic plasticizer is also extensively applied in toys, medical products, printing ink coatings, biodegradable polymers, cosmetics and food additives.^5–8^ Moreover, TBC is a kind of environmental friendly plasticizer because the materials used in its synthesis are available from renewable resources by fermentation processes.^4^ TBC plasticizer would be an entirely bio-renewable and sustainable option because the reaction of citric acid (CA) and butanol from renewable sources has been extensively studied.^1^ Recent biotechnology and bioprocessing advancements on the production of CA and *n*-butanol have been reported in the literature.^9^

The traditional catalysts used in the synthesis of TBC were mainly sulfuric acid, titanate and solid acid.^3,4,8^ The sulfuric acid has many disadvantages such as formation additional by product, corrosion of equipment, complicated manufacturing process and environmental protection issues. The sulfuric acid has one of the important disadvantage that the sulfuric acid cannot be reused. The titanate also has the disadvantages of higher cost, difficult separation from products, and high energy consumption.^3^ The production process of TBC synthesis mainly included the complex processes such as reactive distillation,^2,5^ three stage batch system^7^ and continuous water removal.^10^ Therefore, there was an urgent need to develop environmentally friendly and economical processes.

The solid acid catalysts have been used widely in many reactions because of the obvious advantages such as high acidity, no corrosion of the reactor, lower cost, ease of recovery and reuse.^11,12^ Recently, the solid acid catalysts have been applied in the synthesis of TBC such as ionic liquids,^4^ HZSM-5,^8^ SA/MCM-41 (ref. 13) and USY.^1^ It is well known that heteropoly acids (HPAs) as solid acid has been applied widely in various reaction because HPAs as economically and environmentally solid acid have the advantages of acidity and redox properties.^14,15^ However, industrial use of HPA is limited because of its solubility in polar solvents and small surface area.^16,17^ Hence, when used in heterogeneous catalysis, there is a need to support HPA on suitable solid, which will improve dispersion, acidity and stability.^18^ In the past few decades, polyaniline (PANI) as one of the most important conducting polymers has been extensively studied. Polyaniline (PANI) as one of the important classes of conjugated polymers has been applied widely in many fields because of interesting electrochemical, optical properties and environmental stability.^19^ PANI can be obtained easily by chemical or electrochemical polymerization of aniline in aqueous or non-aqueous media. PANI doped with heteropolyacids as the catalysis has been used in many researchers due to the surface of the active phase of PANI supported catalysts exhibits properties significantly different from those of crystalline catalysts.^20–23^

In this paper, we prepared supported HPA/PANI catalysts, which possesses high active and stable solid acid catalysis, and used them in the synthesis of TBC by esterification of citric acid and butanol to develop sustainable, industrial benign and environmentally friendly catalytic process. The influencing factors of reaction such as HPA loading, the amount of catalyst, molar ratio of citric acid and butanol, reaction temperature time will investigated systematically. Through orthogonal experiment, the optimization of reaction conditions was studied. The reusability of the catalysts was also investigated.

## Experimental

### Preparation of PTA/PANI

Aniline was purchased from Shanghai Sinopharm Chemical Reagent Co. and was purified by distillation under reduced pressure before used. All other chemicals such as phosphotungstic acid (H_3_PW_12_O_40_) was purchased from Shanghai Sinopharm Chemical Reagent Co. and was used without further purification.

The PANI support was prepared by interfacial polymerization. At first, 1.86 g aniline monomer was added in a 100 mL round-bottomed flask. Then, 4.65 g ammonium persulfate dissolved in 50 mL hydrochloric acid solution (1 mol L^−1^) was added slowly into the above system. The reaction was kept standing for 24 h under N_2_ protection in dark conditions. Finally, the product was washed with ethanol and water and dried in a vacuum oven at 60 °C overnight.

0.2 g of phosphotungstic acid was dissolved in 20 mL of water solution. 0.8 g of PANI was slowly added to water solution with string. The resulted mixture was kept overnight for stirring and then washed and dried at 80 °C for 4 h. The supported PTA catalysts prepared in this manner were labelled as 20 wt% PTA/PANI.

### Characterization

Powder X-ray diffraction (XRD) patterns were recorded at ambient temperature on a D/MAX-2400 diffractometer at 40 kV, 100 mA for Cu Kα (*λ* = 1.5418 Å). The metal contents in the catalysts were determined by inductively coupled plasma-atomic emission spectroscopy (ICP-AES) (PerkinElmer Optima 2000DV). The shapes of catalysts were observed by scanning electron microscopy (SEM). FT-IR spectra were characterized by VERTEX70 spectrometer with KBr pellets. In the case of NH_3_-TPD experiments, all catalysts were outgassed in He at 150 °C for 60 min, and finally saturated at 50 °C in a 10% NH_3_/He stream (50 mL min^−1^) for 1 h. After removing most weakly physisorbed NH_3_ by flowing He (50 mL min^−1^) for 30 min, the chemisorbed ammonia was determined by using TCD by heating at 10 °C min^−1^ up to 550 °C under the same flow of He.

### Catalytic performance

The esterification reaction was performed under vigorous stirring in a three-neck glass flask with a water-cooled jacket. 0.45 g citric acid, 0.42 g *n*-butanol and 0.4 g catalyst were added to the reactor. The reaction temperature was slowly raised to reaction temperature and kept constant for reaction time. The liquid reaction feed and product were analyzed by using gas chromatography (GC), GC-7890, capillary column, SPB-5 (30 m length, 0.25 mm width, 0.25 μm diameter) with nitrogen as a carrier gas and a flame ignition detector (FID) in the programmable temperature range of 353 to 553 K. The reaction products were also confirmed by GC-MS (Agilent 6890, HP-5 MS capillary column, 30 m × 0.25 mm × 0.25 μm).

## Results and discussion

**Fig. 1:**
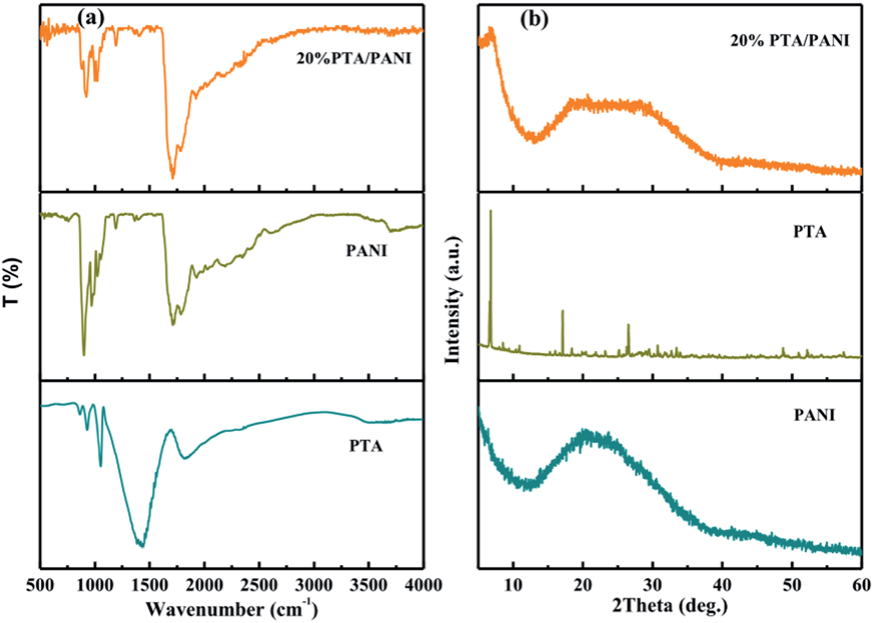
FTIR spectra of PTA, PANI and 20% PTA/PANI (a) and XRD spectra of PTA, PANI and 20% PTA/PANI (b).

The FTIR spectra of PTA, PANI and 20% PTA/PANI were shown in Fig. 1(a). The unsupported PTA shown characteristic absorption bands at 1072, 972 and 900 cm^−1^ those could be interrelated to P–O stretching, terminal W=O stretching and W–O–W asymmetric stretching of corner sharing bridged oxygens, respectively.^18,24,25^Fig. 1 shown the FT-IR spectrum of PANI. The peak at 802 cm^−1^ belonged to the C–H out-of-plane bending vibration of *para*-disubstituted benzene rings. The characteristic band of the dopant anion (HCl-PANI) appeared around 1140 cm^−1^.^26^ The peak centered at 1302 cm^−1^ was due to the C–N stretching mode in Ar–N.^27^ The peak at 1495 and 1576 cm^−1^ belonged to the benzenoid and quinoid C=C stretching vibration of PANI, respectively.^28^ All the above peaks suggest that the HCl doped PANI was successfully synthesized. In supported 20% PTA/PANI catalysts, absorption bands were observed compared with PTA and PANI indicating that supported PTA/PANI catalysts could not change the topological structure of PTA and PANI. The XRD patterns of PTA, PANI and 20% PTA/PANI were shown in Fig. 1(b). PANI exhibited characteristic diffraction pattern of amorphous. After supporting PTA, the XRD patterns of 20% PTA/PANI exhibited diffraction pattern of PANI and PTA indicating the structure was preserved.

**Fig. 2:**
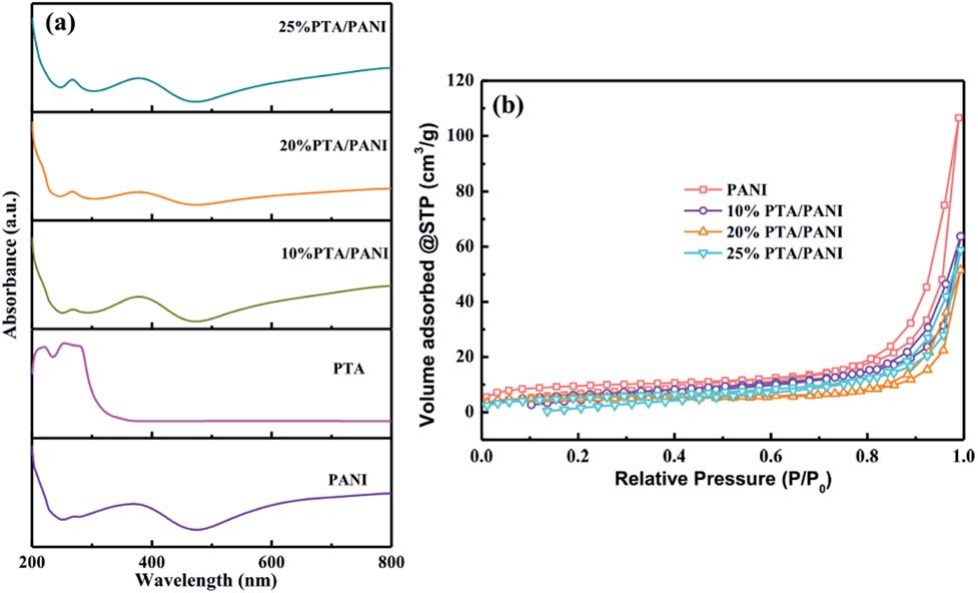
UV-vis spectra of PTA, PANI and PTA/PANI (a) and N_2_ adsorption results (b) for PTA/PANI catalysts.

UV-vis spectra of the prepared PTA, PANI and PTA/PANI were shown in Fig. 2(a). Charge transfer absorption bands of PTA were generally observed between 200–400 nm in UV-vis spectrum.^29–31^ The PTA had strong absorption bands at 260 nm agreement with the previous research.^18^ These bands at 260 could be related to ligand (O) to metal (W) charge transfer transitions involved in edge-sharing and corner-sharing W–O–W bridges present in the Keggin units respectively.^30,31^ A typical absorption spectrum of the polyaniline had distinct absorption bands located 370 nm depending on preparation and/or processing of PANI which the presence of bands in this region had been reported in many researches.^32^ The absorption band at 260 nm was retained in both the supported PTA/PANI samples which shows this particular transition was not affected while supporting PTA on the PANI. The supported PTA catalysts could not affect the structure of PTA which had been reported in previous works.^18^ The N_2_ adsorption isotherms of PTA/PANI were shown in Fig. 2(b) and the specific surface area and porosity are summarized in Table 1. Type I isotherms according to IUPAC classification were observed, a typical feature for materials with microporous structures. The as-synthesized PANI exhibited a Brunauer–Emmett–Teller (BET) surface area of 31 m^2^ g^−1^ and a pore volume of 0.157 cm^3^ g^−1^. A gradual decrease of surface area and pore volume with loaded PTA suggested that the porous PANI were occupied by highly dispersed PTA.

**Table 1:** N_2_ physisorption results of PTA/PANI catalysts

Sample	SA^a^ (m^2^ g^−1^)	MA^b^ (m^2^ g^−1^)	EA^c^ (m^2^ g^−1^)	MV^d^ (cm^3^ g^−1^)
PANI	31	13	18	0.157
10% PTA/PANI	23	10	13	0.098
20% PTA/PANI	17	11	6	0.079
25% PTA/PANI	18	10	8	0.09

a BET surface area.

b Micropore area.

c External surface area.

d Micropore volume.

**Fig. 3:**
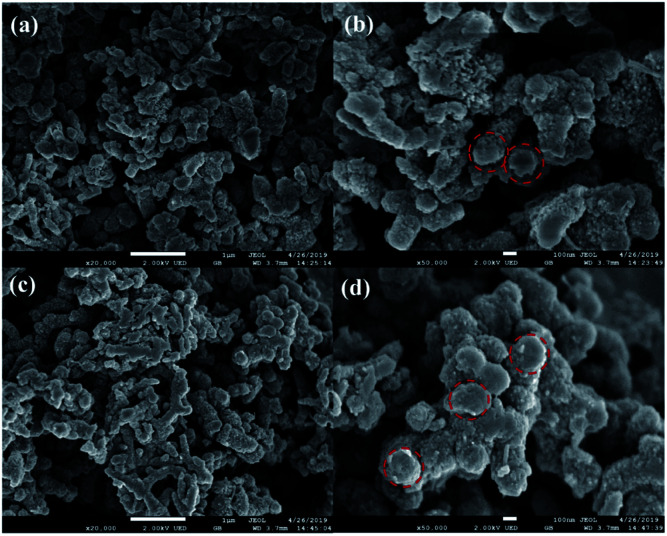
SEM images of PANI (a) and (b) and 20% PTA/PANI (c) and (d).

The representative scanning electron microscope images of PANI and 20% PTA/PANI were presented in Fig. 3. Polymerization in acid led to the granular morphology in Fig. 3 that had often been reported in the literature.^33^ The presence of PANI nanotubes was also observed in the products prepared in solutions of acid in Fig. 3. Nanotubes of 1 μm diameter were produced. The diameter within a single nanotube was relatively uniform but various nanotubes had different thicknesses. Some of the nanotubes were several micrometres long; others were shorter than 300 nm. A similar observation had also been reported for PANI nanotubes.^34^ The morphology of 20% PTA/PANI was consisted with PANI indicating the supported catalysts could not influent the morphology and size of PANI.

**Fig. 4:**
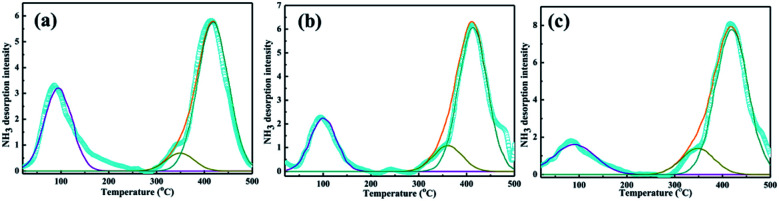
NH_3_-TPD of 10% PTA/PANI (a), 20% PTA/PANI (b) and 25% PTA/PANI (c).

The NH_3_-TPD profiles of PTA/PANI with difference loading of PTA was shown in Fig. 4 and the results were summarized in Table 2. The actual PTA loadings were also summarized in Table 2 which were determined by ICP-OES. The broad desorption peak emerged at 100 °C belonged to weak acid sites. The broad desorption peaks at 300–400 °C corresponded to mediate strong acid sites. The broad desorption peaks at 450 °C belonged to strong acid sites. These acid sites were mainly formed by protons in catalysts. The strong acidity increased resulting from a change in the bonding structure where the nitrogen and oxygen bonding influence the acidity and basicity. The lower dispersion leads monolayer coverage of PTA on the support. However the acid strength was found to increase when the loading was increased due to the formation of multilayer of PTA. Overall a significant increase in total acidity and increase in the intensity of high temperature desorption peaks were observed. With increasing PTA loading, the acid amount and acidic strength of acid site increased significantly.^35,36^ Especially, the PTA/PANI catalyst acidity increased from 0.043 to 0.061 mmol g^−1^ while those of the increasing the loading of PTA from 10% to 20% showed a moderate increase in acidity after increasing the loading of PTA. It was known that the acidity modified shown an important affect the synthesis of TBC.^1,4,8,13^

**Table 2:** NH_3_-TPD data of the catalysts

Sample	HPA loading^a^ (%)	TA^b^ (mmol g^−1^)	WA^c^ (mmol g^−1^)	MA^d^ (mmol g^−1^)	SA^e^ (mmol g^−1^)
10% PTA/PANI	9.2	0.043	0.009	0.005	0.029
20% PTA/PANI	17.6	0.053	0.014	0.003	0.036
25% PTA/PANI	20.8	0.061	0.011	0.008	0.042

a ICP measurement.

b Total acidity.

c Weak acidity.

d Medium acidity.

e Strong acidity.

**Fig. 5:**
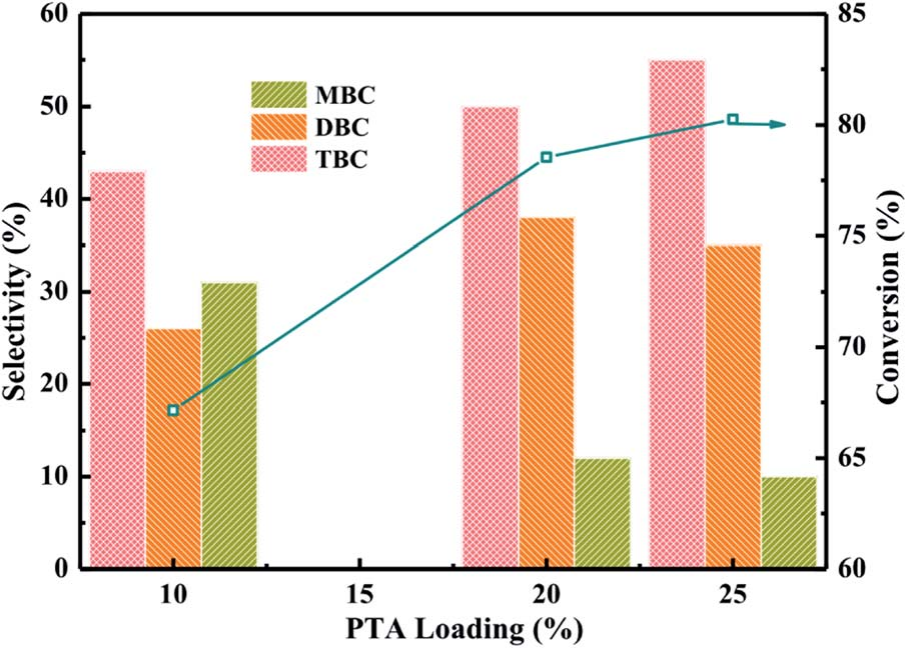
Effect of PTA loading on the conversion of citric acid and selectivity of different products.

The catalytic performance of different loading PTA/PANI catalysts were researched which the model reaction of synthesis TBC by esterification of CA with *n*-butanol was chose. The reaction conditions were molar ratio of CA to *n*-butanol of 1 : 5, CA of 0.2 mol, reaction temperature of 170 °C and reaction time of 6 h. The reaction results were listed in Fig. 5. The reaction products of ester were monobutyl citrate (MBC), dibutyl citrate (DBC) and tributyl citrate (TBC). The conversion of CA found to be increased from 67 to 78% with increase PTA loading from 10% to 20%. The conversion of CA increased to 80% when PTA loading was further increased up to 25%. The overall trend of TBC selectivity obtained was 10% PTA < 20% PTA < 25% PTA which the selectivity of TBC increased with increase the PTA loading. However, the selectivity of MBC decreased with increase the PTA loading. With the increase of PTA loading, MBC might further undergo esterification to produce TBC. All PTA/PANI catalysts showed higher activity and selectivity which might be attributed to the effect of total acidity because the previous research found that the increase of acidity was beneficial to esterification.^1,7,8^ In view to maximize CA conversion, TBC selectivity, the utilization of PTA and the economical of catalyst, the most favourable parameters for esterification were achieved by process optimization over 20% PTA/PANI catalyst because with increase in PTA loading from 20% to 25% on PANI the conversion of CA increased only from 78 to 80%. Therefore, 20% PTA/PANI catalyst was applied in later reactions.

**Fig. 6:**
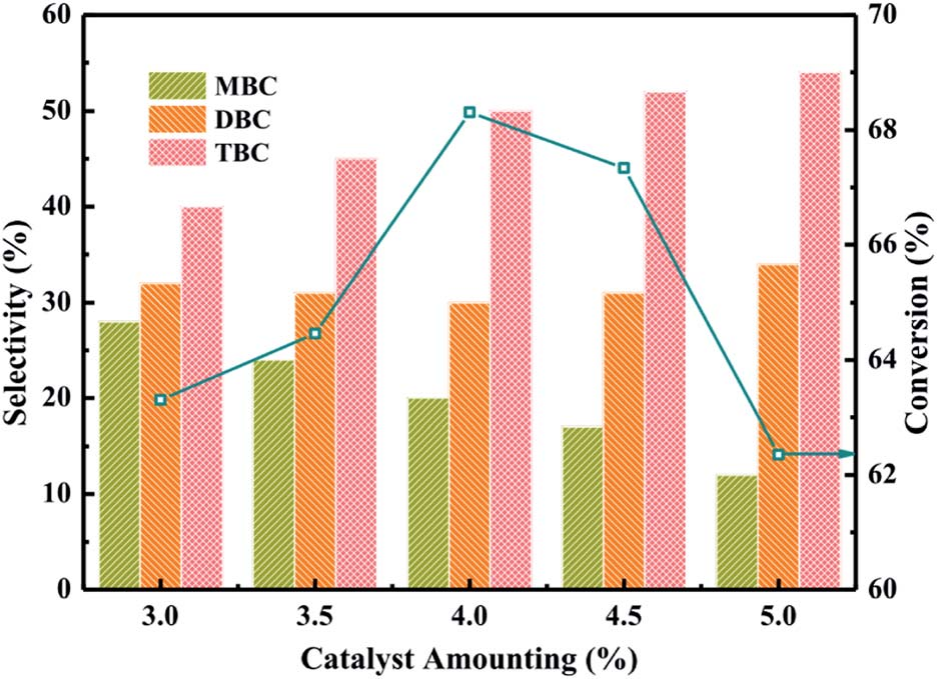
Effect of catalyst amounting on the conversion of citric acid and selectivity of different products.

The catalytic performance of different catalyst amounting were investigated in Fig. 6 which the reaction conditions were molar ratio of CA to *n*-butanol of 1 : 5, CA of 0.2 mol, reaction temperature of 140 °C and reaction time of 3.5 h. The influence of 20% PTA/PANI catalyst amounting on CA conversion and the selectivity of different ester products (MBC, DBC and TBC) was investigated by varying the catalyst amounting from 3% to 5%. The conversion of CA increased from 63 to 68% with increase catalyst amounting from 3% to 4%. The conversion of CA decreased to 62.3% when catalyst amounting further increased from 4% to 5%. The conversion increase could be attributed to proportionally increase in active sites which increases rate of esterification.^37^ However, the conversion decreased with increase the catalyst amounting which could be attributed that a large number of catalysts leaded to the agglomeration of catalysts in the reaction system increasing the diffusion resistance because the density of PANI was light. The selectivity of TBC increased with increase the catalyst amounting. Meanwhile, the selectivity of MBC decreased with increase the catalyst amounting. This might be reasoned that a high catalyst amounting provided a large number of active catalytic sites. Hence, 4% catalyst amounting would be optimum and an appropriate amount at the studied reaction conditions in present study.

**Fig. 7:**
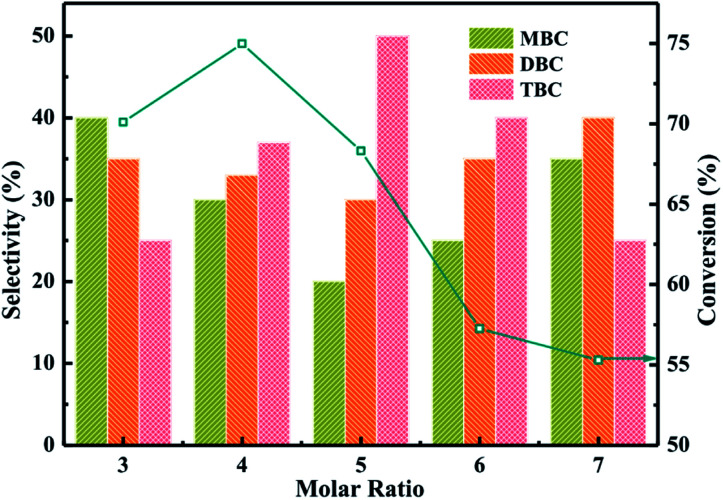
Effect of molar ratio on the conversion of citric acid and selectivity of different products.

The catalytic performance of different molar ratio (*n*-butanol : CA) were investigated for synthesis of TBC by esterification of CA with *n*-butanol in Fig. 7. The reaction conditions were catalyst amounting of 3%, CA of 0.2 mol, reaction temperature of 140 °C and reaction time of 3.5 h. The results obtained at different molar ratio (*n*-butanol : CA) were illustrated in Fig. 7. The conversion of CA increased from 70 to 75% and selectivity of TBC increased from 25 to 35% with increase the molar ratio from 3 to 4. The higher conversion of CA and selectivity of TBC might be attributed that CA occupied the active sites on catalyst surface and the availability of *n*-butanol molecules were utilized for further esterification.^38^ The conversion of CA and selectivity of TBC were observed to be decreased with the further increase of molar ratio of *n*-butanol to CA from 5 to 7. The decrease of TBC selectivity might be due to the *n*-butanol molecules occupied the large active sites on catalyst surface.^1^ The experiment results were agreement with the previous research.^1^

**Fig. 8:**
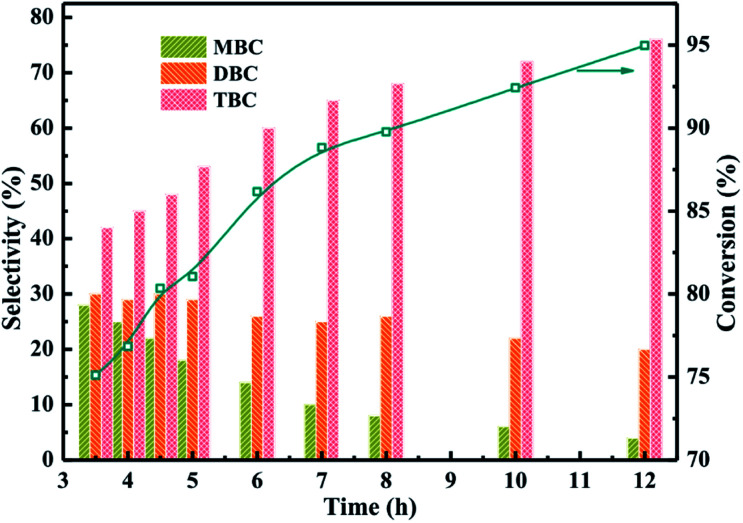
Effect of reaction time on the conversion of citric acid and selectivity of different products.

The catalytic performance of different reaction time were investigated in Fig. 8 which the reaction conditions were molar ratio of CA to *n*-butanol of 1 : 4, CA of 0.2 mol, and reaction temperature of 170 °C. The results obtained at different reaction time from 3.5 h to 12 h were illustrated in Fig. 8. The conversion of CA increased with longer reaction time and the selectivity of TBC was also found to be increased with increasing the reaction time. Meanwhile, the selectivity of MBC and DBC decreased with increase in reaction time which could be attributed to the longer reaction time facilitated MBC and DBC successive esterification formed TBC.^1^

**Fig. 9:**
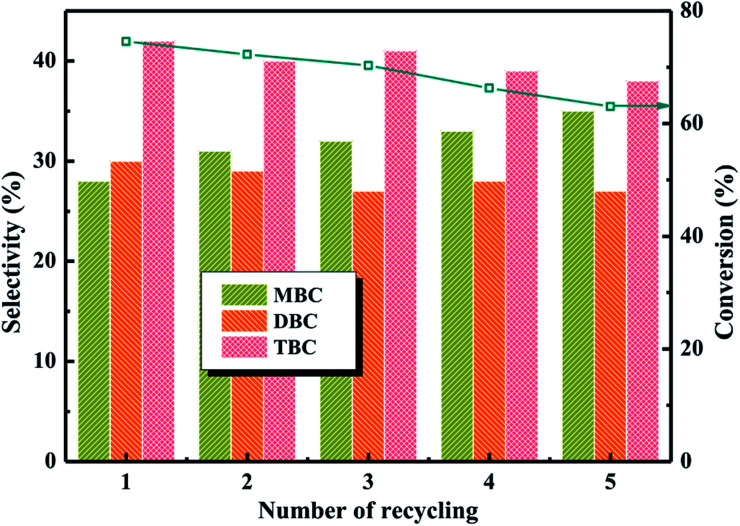
Catalyst recycle studies for 20% PTA/PANI.

The different catalytic tests had shown high conversion and selectivity by using the as-prepared PTA/PANI catalysts for esterification under appropriate conditions. Recyclability, the other essential requirement for industrial catalysts in industrial applications, was evaluated for PTA/PANI with esterification of CA with *n*-butanol. The catalysts can be reused without any reactivation treatments except for a two-time washing with H_2_O between two recycle. The catalytic performance of catalyst recycle were investigated at identical set of reaction conditions: molar ratio of CA to *n*-butanol of 1 : 4, CA of 0.2 mol, reaction temperature of 140 °C and reaction time of 3.5 h in Fig. 9. The recyclability of 20% PTA/PANI for esterification was shown in Fig. 9, which shown that the 20% PTA/PANI exhibited excellent recyclability up to 5 successive cycles. It could be observed that the conversion of citric acid and selectivity of TBC both decrease with increase catalyst recycle number. The conversion of citric acid decreased from 74% to 63% and the selectivity of TBC decreased only from 43% to 39%. The experiment results were agreement with previous research.^8^ This was attributed to the longer catalyst recycle led the catalyst loss decreasing the activity sites.

## Conclusions

The renewable plasticizer of tributyl citrate was synthesized by esterification of renewable citric acid and *n*-butanol over PTA/PANI catalyst. The influence of various process parameters have been investigated over PTA/PANI catalyst with aim to maximize the conversion of CA and the selectivity of TBC. The different catalytic tests have shown that the catalyst shown high conversion and selectivity by using the as-prepared PTA/PANI catalysts for esterification under appropriate conditions. The 20% PTA/PANI catalyst exhibited excellent recyclability up to 5 successive cycles. Our results have revealed that the PTA/PANI catalyst is a promising hetero-catalyst for synthesis of environmental friendly and non-toxic TBC plasticizer from the renewable sources. Further improvements of the selectivity of TBC by optimizing the PTA/PANI catalyst and improving the cyclic stability of PTA/PANI are undergoing in our lab.

## Conflicts of interest

There are no conflicts to declare.

## Supplementary Material
